# Whole Transcriptome Sequencing Reveals Gene Expression and Splicing Differences in Brain Regions Affected by Alzheimer's Disease

**DOI:** 10.1371/journal.pone.0016266

**Published:** 2011-01-21

**Authors:** Natalie A. Twine, Karolina Janitz, Marc R. Wilkins, Michal Janitz

**Affiliations:** 1 School of Biotechnology and Biomolecular Sciences, University of New South Wales, Sydney, New South Wales, Australia; 2 New South Wales Systems Biology Initiative, University of New South Wales, Sydney, New South Wales, Australia; 3 Ramaciotti Centre for Gene Function Analysis, University of New South Wales, Sydney, New South Wales, Australia; Victor Chang Cardiac Research Institute (VCCRI), Australia

## Abstract

Recent studies strongly indicate that aberrations in the control of gene expression might contribute to the initiation and progression of Alzheimer's disease (AD). In particular, alternative splicing has been suggested to play a role in spontaneous cases of AD. Previous transcriptome profiling of AD models and patient samples using microarrays delivered conflicting results. This study provides, for the first time, transcriptomic analysis for distinct regions of the AD brain using RNA-Seq next-generation sequencing technology. Illumina RNA-Seq analysis was used to survey transcriptome profiles from total brain, frontal and temporal lobe of healthy and AD post-mortem tissue. We quantified gene expression levels, splicing isoforms and alternative transcript start sites. Gene Ontology term enrichment analysis revealed an overrepresentation of genes associated with a neuron's cytological structure and synapse function in AD brain samples. Analysis of the temporal lobe with the Cufflinks tool revealed that transcriptional isoforms of the apolipoprotein E gene, *APOE-001*, -*002* and -*005*, are under the control of different promoters in normal and AD brain tissue. We also observed differing expression levels of *APOE-001* and *-002* splice variants in the AD temporal lobe. Our results indicate that alternative splicing and promoter usage of the *APOE* gene in AD brain tissue might reflect the progression of neurodegeneration.

## Introduction

Alzheimer's disease (AD) is the most common cause of dementia in the human population; it mainly affects individuals over the age of 60, and one's risk of developing it increases steadily with age [1]. AD is characterized by a complex progression of neurodegeneration that results in memory impairment and loss of other cognitive processes as well as the presence of non-cognitive symptoms including delusions, agitation and changes in mood and personality. The pathogenesis of AD is complex and remains challenging to research efforts worldwide. The majority of AD cases show no familial or geographical clustering and are described as sporadic or idiopathic. The apolipoprotein E (*APOE*) genotype influences age at onset of AD. Compared to *APOE* e3 (Cys-112, Arg-158), which is considered neutral, the e4 allele (Arg-112, Arg-158) is associated with increased risk and earlier onset of AD in a dose-dependent manner. Conversely, the e2 allele (Cys-112, Cys-158) is protective against AD [2]. In the absence of greater understanding of AD pathogenesis, treatment strategies do not provide a cure but only treat symptoms or reduce the rate of onset [3], [4].

The transcriptome reflects cellular activity within a tissue at a given point in time. Genome-wide expression studies, which are not influenced by deductive assumptions, provide an unbiased approach for investigating the pathogenesis of complex diseases like AD. Transcriptome analyses have been performed using transgenic animals models of AD and patient-derived cell lines [5], [6]. In contrast to these approaches, post-mortem brain tissue is difficult to obtain, and some RNA quality concerns exist that might potentially influence transcriptome studies [7], [8]. Nevertheless, post-mortem brain tissue, being identical to the tissue affected by the disease, remains the gold standard against which all other model systems are evaluated. Transcriptome studies of AD utilizing brain tissue have however generated mostly discordant results. The recent development of next-generation sequencing provides a more comprehensive and accurate tool for transcriptome analysis of this invaluable resource [9], [10].

RNA-Seq analyzes complementary DNA (cDNA) by means of highly efficient, next-generation DNA sequencing methods and subsequent mapping of short sequence fragments (reads) onto the reference genome. That this new technology makes it possible to identify exons and introns, mapping their boundaries and the 5′ and 3′ ends of genes, in turn makes it possible to understand the complexity of eukaryotic transcriptomes comprehensively. Moreover, RNA-Seq enables identification of transcription initiation sites (TSSs) and new splicing variants, and it permits of a precise quantitative determination of exon and splicing isoform expression [11].

Some recent reports, which systematically compare microarrays and next-generation sequencing, have clearly proven the superiority of the latter, both with respect to low frequency of false positive signals and high reproducibility of the method [12], [13]. A recent report by van Bakel et al. concerning transcript analysis of intragenic regions unambiguously showed that hybridization signals from microarrays can lead to massively false positive signals from transcripts of low abundance [14].

In the present study, we performed a comparative gene expression analysis of normal human brain tissue and tissue affected by Alzheimer's disease, using the RNA-Seq technique. Along with samples from whole normal and AD brains, mRNA samples from two different brain regions, namely the frontal and temporal lobes, were analyzed. We found significant differences in gene isoform expression levels, alternated use of promoters and transcription start sites between normal and AD brain tissue.

## Materials and Methods

### Human brain RNA

Total RNA from post-mortem human brains was obtained from Ambion (Austin, USA) and Capital Biosciences (Rockville, USA). Table 1 provides detailed information regarding each sample used in this study. The quality of the total RNA was evaluated using the Agilent 2100 Bioanalyser RNA Nano Chip.

**Table 1:** Source of total RNA from brain tissue samples.

Condition	Sample	Gender	Age (years)	Source
**Normal**	Total brain	13 male;10 female	23–86 (x̃≈68.3)	Ambion
	Frontal lobe	5 male	22–29 (x̃≈26.4)	Capital Biosciences
	Temporal lobe	5 male	23–29 (x̃≈26.0)	Capital Biosciences
**Alzheimer**'**s disease**	Total brain	1 male	87	Capital Biosciences
	Frontal lobe	1 male	87	Capital Biosciences
	Temporal lobe	1 male	80	Capital Biosciences

### Library preparation and sequencing

For the mRNA-Seq sample preparation, the Illumina standard kit was used according to the manufacturer's protocol. Briefly, 10 µg of each total RNA sample was used for polyA mRNA selection using streptavidin-coated magnetic beads, followed by thermal mRNA fragmentation. The fragmented mRNA was subjected to cDNA synthesis using reverse transcriptase (SuperScript II) and random primers. The cDNA was further converted into double stranded cDNA and, after an end repair process (Klenow fragment, T4 polynucleotide kinase and T4 polymerase), was finally ligated to Illumina paired end (PE) adaptors. Size selection was performed using a 2% agarose gel, generating cDNA libraries ranging in size from 200–250 bp. Finally, the libraries were enriched using 15 cycles of PCR and purified by the QIAquick PCR purification kit (Qiagen). The enriched libraries were diluted with Elution Buffer to a final concentration of 10 nM. Each library was run at a concentration of 7 pM on one Genome Analyzer (GAII) lane using 36 bp sequencing. Six samples were analyzed in this manner, taken from frontal, temporal and total brain tissue of both AD and healthy brains.

### Primary processing of Illumina RNA-Seq reads

RNA-Seq reads were obtained using Bustard (Illumina Pipeline version 1.3). Reads were quality-filtered using the standard Illumina process, and a 0 (no) or 1 (yes) was used to define whether a read passed filtering or not. Six sequence files were generated in FASTQ format (sequence read plus quality information in Phred format); each file corresponded to the brain tissue from which the RNA originated. The median number of reads per sequence file (corresponding to one lane on the flow cell) was 14,974,824. The sequence data have been submitted to the NCBI Short Read Archive with accession number SRA027308.2.

### Mapping of RNA-Seq reads using TopHat

Reads were then processed and aligned to the UCSC *H. sapiens* reference genome (build hg19) using TopHat v1.0.12 [15]. TopHat incorporates the Bowtie v0.11.3 algorithm to perform the alignment [16]. TopHat initially removes a portion of reads based on quality information accompanying each read, then maps reads to the reference genome. The pre-built *H. sapiens* UCSC hg19 index was downloaded from the TopHat homepage and used as the reference genome. TopHat allows multiple alignments per read (up to 40 by default) and a maximum of 2 mismatches when mapping reads to the reference. The mapping results were then used to identify “islands” of expression, which can be interpreted as potential exons. TopHat builds a database of potential splice junctions and confirms these by comparing the previously unmapped reads against the database of putative junctions. Default parameters for TopHat were used.

### Transcript assembly and abundance estimation using Cufflinks

The aligned read files were processed by Cufflinks v0.8.0 [17]. Reads were assembled into transcripts, their abundance estimated and tests for differential expression and regulation between the tissue samples were performed. Cufflinks does not make use of existing gene annotations during assembly of transcripts, but rather constructs a minimum set of transcripts that bests describe the reads in the dataset. This approach allows Cufflinks to identify alternative transcription and splicing that are not described by pre-existing gene models [17]. Cufflinks uses the normalized RNA-Seq fragment counts to measure the relative abundances of transcripts. The unit of measurement is Fragments Per Kilobase of exon per Million fragments mapped (FPKM). Confidence intervals for FPKM estimates were calculated using a Bayesian inference method [18].

### Comparison to reference annotation and differential expression testing using Cuffcompare and Cuffdiff

Once all short read sequences were assembled with Cufflinks, the output.GTF files were sent to Cuffcompare along with a reference.GTF annotation file downloaded from the Ensembl database (Homo_sapiens.GRCh37.55.gtf; [19]). This classified each transcript as known or novel. The classification also describes the nature of the match to the reference gene annotation by way of a code letter. These are useful for selecting novel isoforms from the analysis.

Cuffcompare produces a combined.GTF file which is passed to Cuffdiff along with the original alignment (.SAM) files produced by TopHat. Cuffdiff then re-estimates the abundance of transcripts listed in the.GTF file using alignments from the.SAM file, and concurrently tests for differential expression. The expression testing is done at the level of transcripts, primary transcripts and genes. By tracking changes in the relative abundance of transcripts with a common transcription start site, Cuffdiff can identify changes in splicing. Relative promoter use within a single gene is also monitored by following the abundance changes of primary transcripts from that gene. We used Cuffdiff to perform three pairwise comparisons of expression, splicing and promoter use between normal and diseased samples from temporal, frontal and total brain regions.

### Identification of APOE allele in AD samples

To identify which allele of APOE was present in the frontal, temporal lobe and total brain AD samples, the genotype of SNPs rs429358 and rs7412 were determined using the Integrated Genome Viewer.

### Visualization of mapped reads

Mapping results were visualized using both the University of California, Santa Cruz (UCSC) genome browser [20] and a local copy of the Integrative Genomics Viewer software available at http://www.broadinstitute.org/igv/. Views of individual genes were generated by uploading coverage.wig files to the UCSC Genome browser as a custom track. Data files were restricted to the chromosome in question due to upload limits imposed by the genome browser. The same method was used to generate coverage plots for chromosome 1, except here the coverage values were logged (base 2) prior to uploading to the genome browser. This was done to visualize better the full dynamic range of the read coverage.

### Functional analysis of gene lists using DAVID

The Database for Annotation, Visualization and Integrated Discovery (DAVID) v6.7 is a set of web-based functional annotation tools [21]. The functional clustering tool was used to look for functional enrichment for genes over- and under-expressed more than two-fold in Alzheimer's disease. A unique list of gene symbols was uploaded via the web interface, and the background was selected as *Homo sapiens*. Gene Ontology Biological Process was selected as the functional annotation category for this analysis.

### Hardware specifications

TopHat and Bowtie were installed and run on a SGI Altix 4700 64-bit shared memory machine with 1 TB RAM, 128 Dual-Core CPUs of 1.6 GHz. Cufflinks was run on a desktop computer with 4 GB RAM.

## Results

### Analysis of RNA-Seq data

During the amplification step of sequence generation, the Illumina GAII produces clusters of identical sequence fragments. The number of these clusters is reported, as is the percentage that pass quality filtering by the Illumina image analysis software. Across all 6 samples, between 192,093 and 211,779 raw clusters were generated. Between 67.6% and 74.1% of these clusters passed filtering; these values are within the acceptable range recommended by Illumina. The total number of reads produced for each brain sample ranged from 13,442,077 to 15,772,947, with a median of 14,974,824 (Table 2). There was no significant difference in the number of reads from normal and Alzheimer's brain (Student's t-test, p = 0.9). To assess the quality of mapping reads to the reference genome, some key metrics were extracted from the TopHat output and log files, as shown in Table 2. Between 90% and 92% of reads aligned to the reference genome in a unique manner. A small percentage of reads (0.02% to 0.05%) were removed from the analysis prior to mapping to the reference, due to low quality.

**Table 2:** RNA-Seq sequence reads mapping to UCSC Human genome build 19 by TopHat v1.0.12.

	Total brain N^a^	Total brain AD^b^	Temp lobe N	Temp lobe AD	Front lobe N	Front lobe AD
**Total reads**	13,442,077	14,720,816	15,256,752	14,227,702	15,772,947	15,228,832
**Reads removed**	0.05%	0.04%	0.02%	0.04%	0.03%	0.04%
**Unique hits to reference genome**	91.85%	92.42%	92.40%	90.41%	91.46%	90.96%

TopHat allows up to two mismatches when mapping reads to a reference genome. The number of reads removed due to poor quality and the number of reads mapping uniquely to the reference genome are both expressed as percentages of the total number of reads.

a Normal brain samples.

b Alzheimer's disease brain samples.

### Sequence coverage distribution

**Figure 1:**
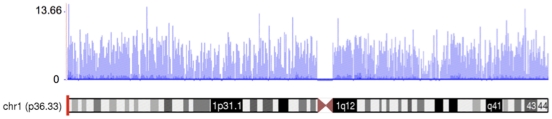
A transcription profile of normal temporal lobe of the brain for chromosome 1. The RNASeq read density along the length of the chromosome is shown. The coverage values are measured along intervals of the genome. These intervals vary in size from 1 bp to 10 Mbp depending on how variable the read density is for a particular genomic location. Each bar represents log_2_ of the frequency reads plotted against chromosome coordinates.

To investigate the level and uniformity of the read coverage against the human genome, we plotted mapped reads of the normal temporal lobe sample along the human chromosome 1 (Fig. 1). We exemplified RNA-Seq coverage on chromosome 1 because this is the largest chromosome in the human karyotype, encoding over 13.6% of all human genes. The coverage values, measured along discrete intervals or bins of the genome, were log-transformed (base 2) to visualize better the full dynamic range of the data. Figure 1 shows the breadth of read coverage across chromosome 1. The read depth in the different bins ranged from 0 to 12,949 and revealed extensive transcriptional activity in the genome. As expected, no reads mapped to the centromere. The total numbers of reads that mapped to chromosome 1 in normal total brain as well as normal temporal and frontal lobes were 1,700,799, 2,062,880 and 2,048,959 respectively.

### Differentially expressed genes

After mapping the RNA-Seq reads to the reference genome with TopHat, transcripts were assembled and their relative abundances calculated using Cufflinks. The summation of FPKM values for every transcript associated with a particular gene gives the expression (abundance) measurement for that gene, in FPKM. Cufflinks uses the Cuffdiff algorithm to calculate differential expression at both the gene and transcript levels. Differential gene expression (DGE) for total brain, frontal and temporal lobes was calculated using the ratio of AD versus normal FPKM values for every gene. The DGE ratios were tested for statistical significance as described recently [22]. The significance scores were corrected for multiple testing using the Benjamini-Hochberg correction.

The range of DGE ratios observed was −26.20 to 26.24 for frontal lobe, −183 to 13.27 for temporal lobe and −350 to 36.63 for total brain. These three ranges for DGE ratios were all statistically significant. The expression ratios in AD versus normal were skewed towards down-regulation. This is potentially due to the lower overall levels of transcriptional activity present in AD vs. normal brain following significant loss of neuronal tissue in the former. The top 10 up- and down-regulated genes in total, frontal and temporal AD brain regions are listed in Tables 3, 4 and 5, respectively.

**Table 3:** Top ten up- and down-regulated genes in AD total brain.

Gene	Description	Chromosome	FPKM N	FPKM AD	Fold change	p-value	Ensembl Gene ID
*IGHA1*	immunoglobulin heavy constant alpha 1	chr14	0.234092	5.275364	22.53543051	0.00018499	ENSG00000211895
*RP11-552E20.3*	not annotated	chr6	1.87539	14.272193	7.610253334	8.76E-009	not annotated
*PCYT1A*	phosphate cytidylyltransferase 1, choline, alpha	chr3	0.413637	3.021956	7.305816453	0.00801203	ENSG00000161217
*SLC7A9*	solute carrier family 7 (cationic amino acid transporter, y+ system), member 9	chr19	0.705822	4.834326	6.849214108	0.0105864	ENSG00000021488
*RAD54L*	RAD54-like (S. cerevisiae)	chr1	0.436495	2.391719	5.479373189	0.0259394	ENSG00000085999
*OAS1*	2′,5′-oligoadenylate synthetase 1, 40/46kDa	chr12	3.82773	20.973536	5.479366622	4.89E-008	ENSG00000089127
*MTIF2*	mitochondrial translational initiation factor 2	chr2	3.75753	16.176999	4.305221515	7.00E-007	ENSG00000085760
*STAB1*	stabilin 1	chr3	0.729626	2.887364	3.9573206	0.0317452	ENSG00000010327
*CD22*	CD22 molecule	chr19	9.83818	36.883742	3.749041184	0	ENSG00000012124
*AC018730.1*	not annotated	chr2	9.4161	32.907895	3.494854027	8.88E-016	not annotated
*RELN*	reelin	chr7	19.4443	0.055404	−350.9548047	2.22E-016	ENSG00000189056
*ANK1*	ankyrin 1, erythrocytic	chr8	13.7202	0.086115	−159.3241596	8.88E-013	ENSG00000029534
*GRM4*	glutamate receptor, metabotropic 4	chr6	29.2203	0.392424	−74.46104214	0	ENSG00000124493
*GRM1*	glutamate receptor, metabotropic 1	chr6	7.96543	0.142632	−55.84602333	1.76E-008	ENSG00000152822
*TFRC*	transferrin receptor (p90, CD71)	chr3	9.17108	0.180114	−50.91819625	3.81E-008	ENSG00000072274
*DAO*	D-amino-acid oxidase	chr12	10.0459	0.20387	−49.27600922	4.99E-008	ENSG00000110887
*ABLIM1*	actin binding LIM protein 1	chr10	19.2058	0.39862	−48.1807235	3.21E-011	ENSG00000099204
*KIAA0802*	KIAA0802	chr18	14.4233	0.387405	−37.23054684	4.61E-007	ENSG00000168502
*MED13L*	mediator complex subunit 13-like	chr12	7.77748	0.210969	−36.86551105	7.40E-010	ENSG00000123066
*ITGB8*	integrin, beta 8	chr7	7.38908	0.20143	−36.68311572	5.17E-007	ENSG00000105855

Differential gene expression for total brain was calculated using the ratio of AD versus normal (N) FPKM values for every gene identified as expressed by Cufflinks. The genes were ranked on their fold changes and the ten with the highest or lowest fold changes are shown here.

**Table 4:** Top ten up- and down-regulated genes in frontal lobe of AD brain.

Gene	Description	Chromosome	FPKM N	FPKM AD	Fold change	p-value	Ensembl Gene ID
*PCK1*	phosphoenolpyruvate carboxykinase 1 (soluble)	chr20	0.121441	3.186619	26.24005896	5.87E-006	ENSG00000124253
*CD163*	CD163 molecule	chr12	0.264139	4.435869	16.79369196	0.000108665	ENSG00000177575
*AC012317.1*	Bac clone	chr16	0.295506	3.913347	13.24286817	0.0128902	not annotated
*NUPR1*	nuclear protein, transcriptional regulator, 1	chr16	7.93458	94.488709	11.90847014	0	ENSG00000176046
*GDPD3*	glycerophosphodiester phosphodiesterase domain containing 3	chr16	0.262915	3.104517	11.80806344	2.23E-006	ENSG00000102886
*STAB1*	stabilin 1	chr3	0.407594	4.278127	10.49604999	0.00152394	ENSG00000010327
*MOV10*	Mov10, Moloney leukemia virus 10, homolog (mouse)	chr1	0.584517	5.521605	9.446440394	0.00022429	ENSG00000155363
*MLKL*	mixed lineage kinase domain-like	chr16	0.268134	2.53291	9.4464335	0.00258816	ENSG00000168404
*LY6G5C*	lymphocyte antigen 6 complex, locus G5C	chr6	0.275959	2.462004	8.921629662	0.00341639	ENSG00000111971
*ITPR3*	inositol 1,4,5-triphosphate receptor, type 3	chr6	0.27173	2.281668	8.396820373	0.00455198	ENSG00000096433
*SLIT1*	slit homolog 1 (Drosophila)	chr10	9.63131	0.367602	−26.20037432	5.71E-006	ENSG00000187122
*PTPRO*	protein tyrosine phosphatase, receptor type, O	chr12	8.77741	0.368512	−23.8185188	1.10E-005	ENSG00000151490
*LPIN2*	lipin 2	chr18	7.50745	0.335313	−22.38937948	1.67E-005	ENSG00000101577
*ATRN*	attractin	chr20	7.2984	0.333062	−21.91303721	1.92E-005	ENSG00000088812
*NAG* (*NBAS*)	neuroblastoma amplified sequence	chr2	6.54659	0.327206	−20.00754876	3.48E-005	ENSG00000151779
*GPR107*	G protein-coupled receptor 107	chr9	7.31149	0.383708	−19.05482815	4.75E-005	ENSG00000148358
*ACOX1*	acyl-CoA oxidase 1, palmitoyl	chr17	8.28587	0.442214	−18.73724034	7.37E-007	ENSG00000161533
*EDEM3*	ER degradation enhancer, mannosidase alpha-like 3	chr1	5.64477	0.303834	−18.57846719	5.57E-005	ENSG00000116406
*ATP8A1*	ATPase, aminophospholipid transporter (APLT), class I, type 8A, member 1	chr4	7.66187	0.412407	−18.57841889	5.57E-005	ENSG00000124406
*VWF*	von Willebrand factor	chr12	6.0129	0.32365	−18.5784026	5.57E-005	ENSG00000110799

Differential gene expression for frontal lobe was calculated using the ratio of AD versus normal (N) FPKM values for every gene identified as expressed by Cufflinks. The genes were ranked on their fold changes and the ten with the highest or lowest fold changes are shown here.

**Table 5:** Top ten up- and down-regulated genes in temporal lobe of AD brain.

Gene	Description	Chromosome	FPKM N	FPKM AD	Fold change	p-value	Ensembl ID
*AC074289.1*	Bac clone – not annotated	chr2	0.28593	3.793698	13.26792572	0.0129943	not annotated
*MT1G*	metallothionein 1G	chr16	15.1637	148.115649	9.767777587	0	ENSG00000125144
*S100A4*	S100 calcium binding protein A4	chr1	3.0191	23.552175	7.801058262	4.44E-016	ENSG00000196154
*DES*	desmin	chr2	4.23774	31.344441	7.396499313	0	ENSG00000175084
*C19orf42*	UPF0608 protein C19orf42 Precursor	chr19	0.626087	4.153445	6.633974192	0.0132315	ENSG00000214046
*MTPAP*	mitochondrial poly(A) polymerase	chr10	1.87181	12.003598	6.412829294	0.000124237	ENSG00000107951
*NME3*	non-metastatic cells 3, protein expressed in	chr16	9.40287	45.776586	4.86836317	0	ENSG00000103024
*KIF1C*	kinesin family member 1C	chr17	39.0482	180.483489	4.622069366	0	ENSG00000129250
*MAP4K4*	mitogen-activated protein kinase kinase kinase kinase 4	chr2	5.65184	24.058735	4.256796902	7.85E-009	ENSG00000071054
*TGFB3*	transforming growth factor, beta 3	chr14	4.62642	17.951668	3.880250388	0	ENSG00000119699
*MICAL2*	microtubule associatedmonoxygenase, calponin and LIM domain containing 2	chr11	43.9961	0.240419	−182.9976	0	ENSG00000133816
*DYNC1I1*	dynein, cytoplasmic 1, intermediate chain 1	chr7	51.4985	0.292	−176.364726	2.96E-013	ENSG00000158560
*RPH3A*	rabphilin 3A homolog (mouse)	chr12	42.8148	0.271284	−157.8227982	9.50E-013	ENSG00000089169
*RASGRF1*	Ras protein-specific guanine nucleotide-releasing factor 1	chr15	29.1194	0.19051	−152.8497192	1.32E-012	ENSG00000058335
*ATP2B1*	ATPase, Ca++ transporting, plasma membrane 1	chr12	27.8105	0.195853	−141.9968037	2.82E-012	ENSG00000070961
*ELMOD1*	ELMO/CED-12 domain containing 1	chr11	25.7148	0.185023	−138.9816401	0	ENSG00000110675
*NELL2*	NEL-like 2 (chicken)	chr12	48.256	0.356889	−135.2129093	4.64E-012	ENSG00000184613
*PDE2A*	phosphodiesterase 2A, cGMP-stimulated	chr11	33.2491	0.250937	−132.4997908	5.69E-012	ENSG00000186642
*CAMKK2*	calcium/calmodulin-dependent protein kinase kinase 2, beta	chr12	44.693	0.352967	−126.6209022	8.97E-012	ENSG00000110931
*ICAM5*	intercellular adhesion molecule 5, telencephalin	chr19	20.7834	0.170851	−121.6463468	1.34E-011	ENSG00000105376

Differential gene expression for temporal lobe was calculated using the ratio of AD versus normal (N) FPKM values for every gene identified as expressed by Cufflinks. The genes were ranked on their fold changes and the ten with the highest or lowest fold changes are shown here.

When comparing the top 30 most over- and under-expressed genes in AD across the 3 brain samples (Tables S1, S2, S3), *DHX58* (DEXH box polypeptide 58) and *STAB1* (Stabilin 1) are up-regulated in both total brain (2.13 fold change (FC), p = 0.01 and 4.9 FC, p = 0.01, respectively) and frontal lobe (3.96 FC, p = 0.03 and 10.5, p<1×10^−16^, respectively), while *TFR1* (transferrin receptor) is down-regulated in both regions (−50.92 FC, p = 3.8×10^−8^ and -17.15 FC, p = 9.2×10^−5^, respectively). *SLIT1* (slit homolog 1) is down-regulated in both frontal and temporal lobes (−26.2 FC, p = 5.7×10^−6^ and −116.67, p = 2×10^−11^). TFR1, responsible for cellular uptake of iron, has been implicated in neurologic development in mice, and accumulation of iron in brain-specific regions has been implicated in AD [23], [24]. SLIT1 is widely reported to be involved in brain development and axon guidance [25].

In the top 30 over- and under-expressed genes in AD between the 3 brain samples, there are a number of genes without annotation, described either as putative or novel transcripts in the Ensembl database. RP11-552E20.3 and AC018730.1 are up-regulated in AD total brain (7.61 FC, p = 8.76×10^−9^ and 3.49 FC, p = 8.88×10^−16^, respectively), AC074289.4 is up-regulated in AD temporal lobe (13.27 FC, p = 0.01) and RP4-697K14.12 is up-regulated in AD frontal lobe (5.77 FC, p = 0.02). None of these putative or novel transcripts is described as protein coding by Ensembl.

There is some concordance between gene expression differences found with RNA-Seq and those reported in previous microarray studies on Alzheimer's disease [9]. Genes in the AD temporal lobe detected as down-regulated by both approaches include dopamine receptor 2 (*DRD2*), AMPA1 receptor (*GRIA1*), glutamate receptor, ionotropic, N-methyl D-aspartate 1 (*GRIN1*), glutamate transporter EAAT3 (*SLC1A1*), a-synuclein (*SCNA*), high affinity BDNF/NT-3 receptor (*TrkB*), high affinity NT-3 receptor (*TrkC*), glutamic acid decarboxylase 1 (*GAD1*) and glutamic acid decarboxylase 2 (*GAD2*). There is also concordance in genes expressed in the frontal lobe, where *DNM1* and *SYN2* are down-regulated, in both our data and previous microarray studies. A comparison also highlights some contradicting results, however, between RNA-Seq and microarray techniques. *PPP3CB* is up-regulated in the temporal lobe in the microarray study [26] but down-regulated in our dataset. *GRIA4* and *GRIK1* are shown to be expressed in senile plaques (in temporal lobe) in microarray data [27] but are not identified as expressed in the AD temporal lobe in the present RNA-Seq dataset.

### Gene Ontology term enrichment analysis of differentially expressed genes

The NCBI web-based functional annotation tool DAVID v 6.7 (Database for Annotation, Visualization and Integrated Discovery) was used to investigate functional associations of gene expression changes seen in AD brain [21]. Genes that were more than two-fold over- or under-expressed were analyzed by functional clustering. Gene Ontology Biological Process was selected as the annotation category for clustering. Once the tool has identified enriched ontologies for a particular gene list, it clusters those that have a statistically significant overlap in terms of their constituent genes. The gene lists used in this analysis contained 1416, 1071 and 944 genes for temporal, whole and frontal brain samples, respectively.

There is a high degree of overlap between the top ten most enriched clusters (Tables S4, S5, S6). Protein localization is the most enriched cluster across all three regions, while vesicle mediated transport and phosphate metabolic processes are within the top five clusters and proteolysis and regulation of GTPase activity are within the top seven for all three tissue samples. The only brain-specific cluster present in the top ten across all three samples is neuronal development. This level of functional overlap between the samples is to be expected given that they all originate from the same tissue.

Interestingly, the frontal lobe is different from the other samples in that it shows greater changes in genes associated with brain-specific biological processes. These are regulation of synaptic transmission (rank 9), neurotransmitter transport (11), response to metal ion (13), metal ion transport (15), regulation of synaptic plasticity (18), negative regulation of neuron apoptosis (19) and axon transport (20). By contrast, the brain-specific categories apparent in the temporal lobe are axon transport (rank 14) and neurotransmitter transport (18), and cerebellum development (12) is implicated for the total brain.

Genes known to be involved in programmed cell death were enriched in the frontal lobe of AD brain (rank 10) and an induction of apoptosis is present in both frontal and temporal lobes (rank 16 and 12, respectively). An over-representation of apoptosis-related genes clearly indicates the ongoing process of neurodegeneration and associated cell loss. The top 20 DAVID functional clusters for total, frontal and temporal brain regions can be seen in Tables S4, S5 and S6, respectively.

### Alternative splicing and transcript identification using RNA-Seq

A key feature of RNA Seq is its ability to identify alternative splicing of transcripts. It also has an advantage over microarray-based methods of detection in its ability to identify novel transcripts. Accordingly, we next investigated the splicing status of all genes and whether genes show differential splicing patterns between normal and diseased tissues.

TopHat builds a database of potential splice junctions by identifying the splice donor and acceptor sites (GT-AG) for each region of a gene with high coverage of short mRNA reads. TopHat then compares the previously unmapped reads against this database of putative junctions. Regions of genes with a high coverage are also screened for internal junction sites. One of the advantages of identifying potential exons without using predefined annotation information is the capability to highlight splicing in unannotated regions of the genome.

A range of 52,438 to 54,808 splice junctions was predicted for normal brain (Table 6). This corresponds to 2.1–2.2% of all reads. By contrast, AD brain samples showed a lower number of splice variants, ranging from 17,265 to 29,012 predicted junctions. This corresponds to 0.47–1.28% of all reads. This difference is statistically significant (Student's t-test, p = 0.043).

**Table 6:** Splice junctions in normal and Alzheimer's brains predicted by TopHat.

	Total brain N^a^	Total brain AD^b^	Temp lobe N	Temp lobe AD	Front lobe N	Front lobe AD
**Total reads**	13,442,077	14,720,816	15,256,752	14,227,702	15,772,947	15,228,832
**Total splice junctions**	54,458	29,012	52,438	17,265	54,808	38,647
**Reads mapping to splice junctions (%)**	2.14%	0.94%	2.10%	0.47%	2.20%	1.28%

RNA-Seq data were mapped to the UCSC Human genome build 19. The number of splice junctions predicted by TopHat is shown, as well as the percentage of the total number of reads.

a Normal brain samples.

b Alzheimer's disease brain samples.

Using the Cuffdiff algorithm to calculate differential expression at the transcript level allowed discovery of which transcripts are common, differentially expressed or present/absent between normal and AD brain tissue.

**Figure 2:**
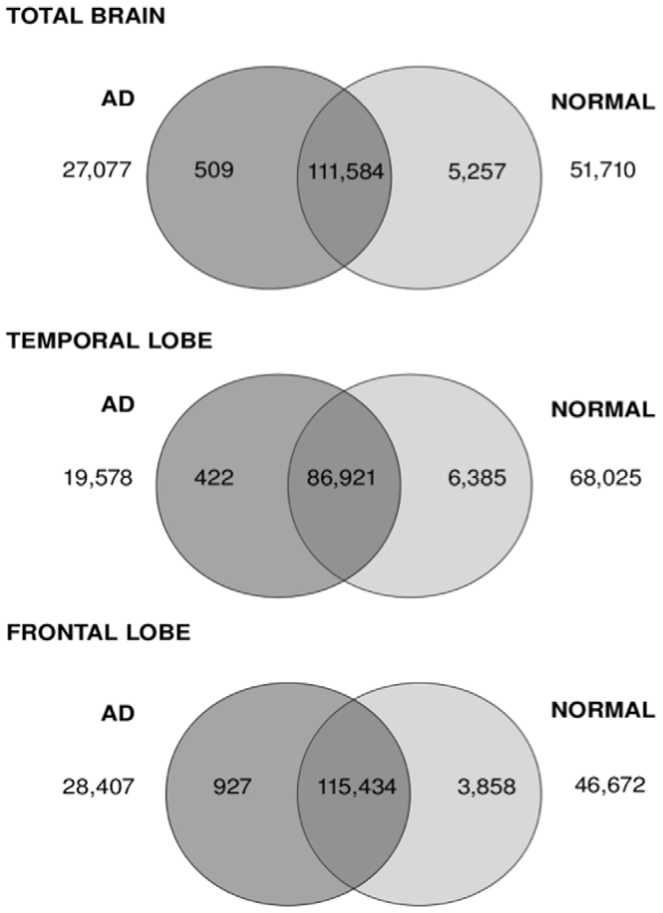
Venn diagram showing distributions of differentially expressed transcripts between healthy and AD brain. Venn diagram showing the number of differentially expressed transcripts between AD and normal tissue samples across total brain, temporal and frontal lobe. The number of transcripts unique to AD and normal tissues is shown in universe area outside the circles. The numbers of transcripts up-regulated by more than two-fold in AD tissue are indicated in the dark grey circle, while the numbers up-regulated by more than two-fold in normal tissue are highlighted in the light grey circle. The intersection of the two circles refers to number of transcripts which are expressed in both AD and normal tissues but which are less than two-fold different in expression level.

Frontal, temporal and total brain specimens showed a large proportion of transcripts at similar expression levels between normal and AD tissue (Fig. 2). Specifically, there were 56%, 48% and 59% of transcripts showing less than two-fold expression difference in the total brain, temporal and frontal lobes, respectively. The number of transcripts up-regulated in AD tissue as compared to normal brain ranged from 422 to 927, representing 0.2–0.5% of total transcripts. The number of transcripts up-regulated in normal tissues compared to AD brain was larger in each case, ranging from 3858 (1.98%) to 6385 (3.52%).

Further analysis revealed a considerable portion of transcripts that were unique to either AD or normal brains. AD brain tissue showed between 19,578 and 28,407 (10.7–14.5%) unique transcripts compared to the corresponding normal tissue. Larger numbers of transcripts were seen to be unique to normal tissue, for which between 46,672 to 68,025 transcripts were observed (23.9% to 37.5%).

### Transcriptional and post-transcriptional regulation between normal and AD brain tissue

To detect transcriptional regulation, RNA-Seq data can be analyzed with Cufflinks. This identifies how many transcription start sites (TSS) are used in each gene and groups transcripts from that gene by their TSS. Each TSS is thus associated with a primary transcript. Cufflinks compares ratios of grouped transcripts between normal and AD tissue to detect alternative promoter usage. Cufflinks also identifies post-transcriptional regulation by looking for changes in relative abundances of mRNAs spliced from the same primary transcript between normal and AD tissue, which it detects as alternative splicing. In this way, Cufflinks discriminates between transcriptional and post-transcriptional processing [17].

Cufflinks analysis of the transcriptome from total brain, temporal and frontal lobe samples revealed that numerous genes are controlled by different promoters in normal and AD tissue (Table 7). Comparative analysis of the total brain samples resulted in the identification of five genes (*CANX*, *DNAJC5*, *MGEA5*, *TMEM66*, *WDR92*) with statistically significant usage of alternative promoters in AD samples (p<0.05 and passing false discovery rate threshold). Using the same selection criteria, frontal and temporal lobe samples from the AD brain showed alternative promoter usage in eleven genes (*ACAP3*, *ARGLU1*, *CHD3*, *KIF5A*, *LENG8*, *MAPK3*, *NR1D1*, *PDE1B*, *PIP5K2B*, *RPH3A*, *WDR47*) and three genes (*APOE*, *KIF5A*, *PP2R4*), respectively.

**Table 7:** Genes showing alternative promoter usage.

Gene	Description	p-value
**Total brain**		
*CANX*	calnexin	0
*DNAJC5*	DnaJ (Hsp40) homolog, subfamily C, member 5	5.64E-006
*MGEA5*	meningioma expressed antigen 5 (hyaluronidase)	0
*TMEM66*	transmembrane protein 66	1.16E-009
*WDR92*	WD repeat domain 92	0
**Frontal lobe**		
*ACAP3*	ArfGAP with coiled-coil, ankyrin repeat and PH domains 3	2.24E-005
*ARGLU1*	arginine and glutamate rich 1	6.43E-007
*CHD3*	chromodomain helicase DNA binding protein 3	0
*KIF5A*	kinesin family member 5A	2.35E-013
*LENG8*	leukocyte receptor cluster (LRC) member 8	0
*MAPK3*	mitogen-activated protein kinase 3	0
*NR1D1*	nuclear receptor subfamily 1, group D, member 1	0
*PDE1B*	phosphodiesterase 1B, calmodulin-dependent	0
*PIP5K2B*	phosphatidylinositol-5-phosphate 4-kinase, type II, beta	2.22E-016
*RPH3A*	rabphilin 3A homolog (mouse)	0
*WDR47*	WD repeat domain 47	0
**Temporal lobe**		
*APOE*	apolipoprotein E	1.92E-006
*KIF5A*	kinesin family member 5A	0
*PPP2R4*	protein phosphatase 2A activator, regulatory subunit 4	7.18E-007

Genes identified by Cufflinks as exhibiting statistically significant alternative promoter usage between normal and AD tissue. Results are shown for total brain, frontal and temporal lobe tissue.

We also investigated whether splicing patterns for transcripts sharing the same transcription start site (TSS) differ between normal and AD brain tissue (Table 8). Statistically significant alternative splicing between normal and AD total brain was detected for the following four genes: *CALM3*, *CANX*, *DNAJC5* and *MGEA5.* Moreover, alternative splicing was detected at a statistically significant level in frontal and temporal brain samples for fifteen and four genes, respectively. For the frontal lobe these include *ACAP3*, *AP2B1*, *ATN1*, *B2M*, *CHD3*, *CTBP1*, *EFHD2*, *LENG8*, *MAPK3*, *NR1D1*, *NUDCD3*, *PDE1B*, *RHBDD2*, *SEPT5* and *WDR47*, and the genes *APOE*, *KIF5A*, *PDZD4* and *SPTBN1* in the temporal lobe.

**Table 8:** Genes showing alternative splicing.

Gene	Description	p-value
**Total brain**		
*CALM3*	calmodulin 3 (phosphorylase kinase, delta)	1.11E-016
*CANX*	calnexin	0
*DNAJC5*	DnaJ (Hsp40) homolog, subfamily C, member 5	1.38E-008
*MGEA5*	meningioma expressed antigen 5 (hyaluronidase)	0
**Frontal lobe**		
*ACAP3*	ArfGAP with coiled-coil, ankyrin repeat and PH domains 3	0
*AP2B1*	adaptor-related protein complex 2, beta 1 subunit	1.07E-010
*ATN1*	atrophin 1	2.34E-008
*B2M*	beta-2-microglobulin	6.68E-004
*CHD3*	chromodomain helicase DNA binding protein 3	3.16E-005
*CTBP1*	C-terminal binding protein 1	1.06E-009
*EFHD2*	EF-hand domain family, member D2	2.66E-007
*LENG8*	leukocyte receptor cluster (LRC) member 8	0
*MAPK3*	mitogen-activated protein kinase 3	0
*NR1D1*	nuclear receptor subfamily 1, group D, member 1	3.73E-007
*NUDCD3*	NudC domain containing 3	2.11E-004
*PDE1B*	phosphodiesterase 1B, calmodulin-dependent	3.42E-004
*RHBDD2*	rhomboid domain containing 2	0
*SEPT5*	septin 5	4.44E-016
*WDR47*	WD repeat domain 47	6.65E-009
**Temporal lobe**		
*APOE*	apolipoprotein E	1.56E-010
*KIF5A*	kinesin family member 5A	2.22E-016
*PDZD4*	PDZ domain containing 4	9.39E-005
*SPTBN1*	spectrin, beta, non-erythrocytic 1	8.47E-007

Gene names for transcripts identified by Cufflinks as exhibiting statistically significant alternative splicing between normal and AD tissue. Results are shown for total brain, frontal and temporal lobe tissue. Alternative splicing is detected between transcripts, which share the same transcription start site (TSS).

### Identification of alternative splicing and promoter usage for apolipoprotein E (APOE)

**Figure 3:**
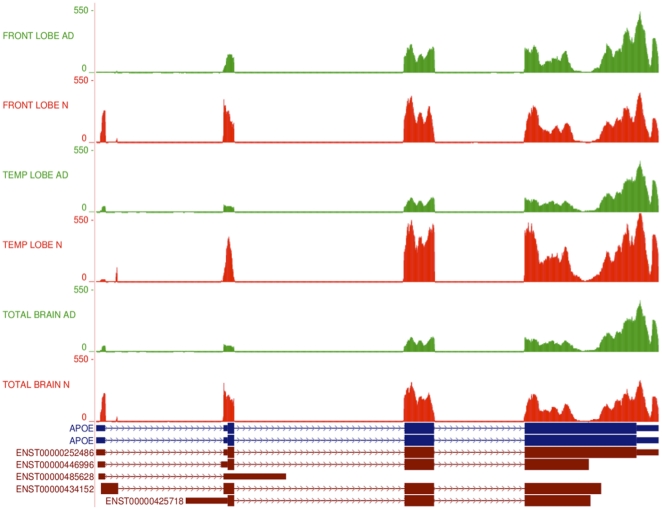
RNA-Seq read mapping to the reference for *APOE.* RNA-Seq read mapping to the UCSC reference genome (hg19) of the gene *APOE* for all 6 samples in this study. The AD tracks are shown in green and normal samples in red. It is clear that the reads map to the 4 exons of the *APOE* gene as annotated in the UCSC database (*APOE* exons shown in blue). The absolute read counts for each sample are indicated on the y axis. A schematic representation of the 5 Ensembl transcripts for *APOE* is shown in brown at the bottom of the figure. N – normal brain samples; AD – Alzheimer's disease brain samples.

**Figure 4:**
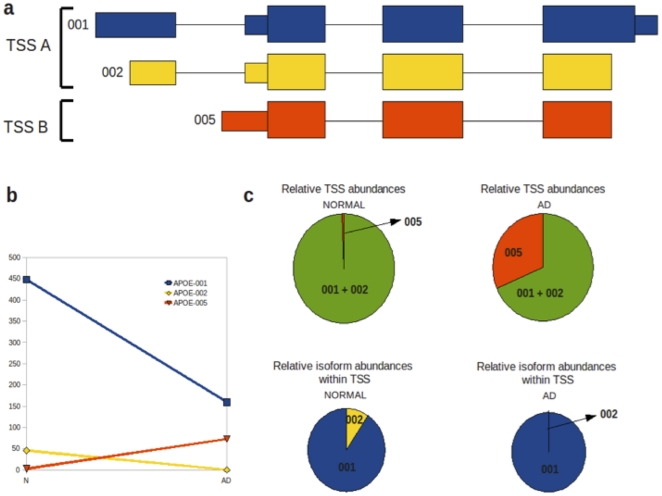
Alternative splicing and promoter usage for the *APOE* gene in temporal lobe tissue. (**a**) Transcriptional isoforms *APOE-001*, *APOE-002* and *APOE-005* are detected in both normal and AD temporal lobes; APOE-001 and -002 have transcription start site (TSS) A and 005 is initiated at TSS B. Isoform 005 comprises exons 2, 3 and 4 while isoforms 001 and 002 contain all 4 exons. (**b**) Isoforms 001 and 002 show decreased expression in AD relative to normal temporal lobe, while isoform 005 shows a relative increase in the AD temporal lobe. (**c**) Relative changes in TSS abundance between normal and AD temporal lobes are indicated by the green/red pie charts, while changes in the two TSS A group isoforms (001 and 002) between normal and AD temporal lobes are shown by the blue/yellow pie charts.

Apolipoprotein E gene (*APOE*) is of particular interest due to its relevance to AD molecular pathology [28]. The mapping of reads for all six samples to the reference genome shows differences in expression levels for individual *APOE* exons (Fig. 3). Cufflinks quantification of differential gene expression showed a 2.13-fold down-regulation of *APOE* in the AD temporal lobe (p = 4.19×10^−7^). It also highlighted the possibility of differential gene splicing. Detailed analysis of transcripts revealed three different *APOE* transcriptional isoforms, namely *APOE-001* (ENST00000252486), *APOE-002* (ENST00000446996) and *APOE-005* (ENST00000425718), in both temporal lobe samples. The *APOE-001* and *-002* isoforms contain exon 1 whereas the *-005* isoform is generated by an alternative promoter upstream of the second *APOE* exon. Two transcription start sites (TSS) were identified for the *APOE* gene in both temporal lobe samples, which will be referred to as TSS A and TSS B. Isoforms *APOE-001* and *-002* are transcribed from TSS A, while *APOE-005* is transcribed from TSS B (Fig. 4a). Comparative analysis of TSS A and TSS B revealed a 26.5-fold up-regulation of the latter in AD temporal lobe (p<1×10^−16^) and 3.09-fold down-regulation of the former in AD temporal lobe (p = 5.11×10^−15^; Fig. 4b,c).

In addition to a switch in promoter usage in the normal and AD temporal lobe, significant alternative splicing between the two isoforms is seen under the control of TSS A (p = 1.46×10^−10^). The abundance of isoform *APOE-002* is reduced in AD temporal lobe to an almost negligible level of 0.02 FPKM, compared with 45.83 in the normal counterpart. *APOE-001* also shows a reduction in abundance of 2.81-fold in AD relative to normal temporal lobe, however it still remains the dominant isoform expressed in the AD temporal lobe at 159.43 FPKM. The *APOE-005* isoform has a FPKM of 73.08 in the AD temporal lobe (Fig. 4c).

A comparison of *APOE* splicing and promoter use in the frontal lobe and total brain did not reveal expression pattern differences as seen in the temporal lobe. Cufflinks does not detect the *APOE-002* isoform in either frontal or total brain samples, and no alternative splicing or promoter usage was detected between the normal and AD samples. Focusing on *APOE* expression in temporal lobe clearly illustrates that, used together, RNA-Seq and Cufflinks can identify not only transcriptional regulation of a gene but also post-transcriptional regulation of primary transcripts via alternative splicing.

### Identification of APOE alleles in the AD samples

To identify which allele of *APOE* was present in the temporal, frontal lobe and total brain AD samples, the genotype of SNPs rs429358 and rs7412 were determined. These two SNPs are associated with the amino acid changes at positions 112 and 158 in the ApoE isoforms. SNP rs429358 showed a T/T genotype for temporal, frontal lobe and total brain samples. This genotype translates to a Cys at position 112 of the protein. SNP rs7412 showed a C/C genotype in temporal lobe and total brain samples and a C/T genotype for the frontal lobe sample. The C allele translates to an Arg at position 158 of the protein, while a T allele translates to a Cys. The Cys112/Arg158 combination in the ApoE protein reflects the presence of the e3 allele, while the Cys112/Cys158 combination indicates presence of the *APOE* e2 allele. Thus, both temporal lobe and total brain AD samples exhibit the *APOE* e3 allele, while frontal lobe AD sample has an equal mix of the e2 and e3 allele.

## Discussion

Our study provides the first comprehensive insight into the transcriptome of brain tissue affected by Alzheimer's disease. Using a whole transcriptome sequencing technique (RNA-Seq), we were able to identify the levels of differentially expressed genes and establish genes with alternative promoter usage and splicing patterns that changed in association with neurodegeneration. Moreover, comparative analysis of samples derived from different brain regions produced an increased molecular resolution for our analysis. This revealed that the frontal and temporal lobes of AD brains not only differed in the quantitative composition of the genes expressed but also showed lobe-specific alternations in transcript assembly.

For whole transcriptome sequencing, we used an Illumina Genome Analyser II with 36 bp sequence reads length. We obtained ∼14×10^6^ sequence reads per sample, which has been previously reported to deliver sufficient sequence coverage for transcriptome profiling [13]. Our rate of 90-92% of reads that map to the reference genome met quality standards of the RNA-Seq technique [29]. An estimation of the number of reads covering chromosome 1 (1,937,546 reads on average) was approximately 12.9% of all reads generated per transcriptome (14,974,824 reads on average). Human chromosome 1 comprises 8% of the human genome and contains 3,141 genes, or 13.6% of all annotated genes [30]. Hence, we conclude that our mRNA-Seq data provide good representation of expressed genes in the human genome.

Cufflinks analysis of gene isoform expression levels, alternative splicing and alternative promoter usage revealed significant differences in transcriptome profiles between frontal and temporal lobe of the AD brain. These variations might reflect temporal and spatial differences in the progression of AD neuropathology across the aging brain. Widespread neuronal loss and a presence of the intraneuronal neurofibrillary tangles (NFTs) and the extracellular neuritic or senile plaques (NPs) are key features of the AD neuropathology. The main components of NPs are peptides of varying length collectively described as beta-amyloid whereas NFTs are mainly composed of paired helical filaments of a hyperphosphorylated form of the microtubule-associated protein tau (MAPT) [31], [32]. NFTs first arise in the entorhinal cortex of the medial temporal lobe and then spread toward the hippocampal CA1 region. NFTs formation then progresses to the temporal and frontal neocortices, and finally affects primary cortices [33]. Thus the temporal and frontal lobe samples used in this study might approximately represent brain regions at distinct stages of the neurodegeneration process, with the temporal lobe affected first, followed by the frontal lobe of the brain.

The tissue-specific enrichment for gene ontology processes suggest region-specific, sequential progression of brain tissue neurodegeneration, with the temporal lobe being affected earlier than the frontal part of the cortex [33]. Consequently, neuronal activity in the frontal lobe may be more vigorous at the time of sample donation. This might count for over-representation of GO terms such as regulation of synaptic plasticity and negative regulation of neuronal apoptosis. In contrast, neurons of the temporal lobe might exist in a more advanced phase of functional deterioration. This in turn is reflected by the more non-neuronally specific transcriptome patterns seen in samples derived from the total brain in this study. We do observe an over-representation of genes related to apoptosis that is consistent with previous reports, however there was no evidence in our analysis for AD-associated changes in the immune response [34].

Many of the changes we observed in gene expression between normal and AD brains were similar to those reported previously. However, some differences were noted. This lack of concordance among our RNA-Seq transcriptome data set and previously reported gene expression profiles is likely to stem from inherent limitations in microarray systems. For example, background levels of hybridization (i.e. hybridization to a probe that occurs irrespective of the corresponding transcript's expression level) limit the accuracy of microarray expression measurements, particularly for transcripts present at low abundance. Furthermore, probes differ considerably in their hybridization properties [35]. Thus, although comparing hybridization results across arrays can identify gene expression differences among samples [36], hybridization results from a single sample may not provide a reliable measure of relative expression for different transcripts. By contrast, the Illumina sequencing data have been described as replicable with relatively little technical variation, thus for many purposes it may suffice to sequence each mRNA sample only once. The information gained from a single lane of Illumina flow cell, as done in the present study, provides a comprehensive analysis of transcripts and enables identification with confidence of differentially expressed genes [11], [37].

Moreover, validation techniques such as quantitative PCR (qPCR) [38], [39] and spike-in RNA [29] have demonstrated that RNA-Seq is extremely accurate. Accordingly, a false positive rate <2% has been demonstrated for this technique [40]. As recently reported by Marioni et al., qPCR results agreed more closely with Illumina sequencing results than with microarrays [11].

Regarding quantification of gene expression, Cufflinks analysis of RNA-Seq data allowed us to dissect expression of individual genes into quantification of particular mRNA isoforms contributing to the final cumulative value of gene expression. To our knowledge, this is the first report where quantitative information about particular splice variants at a genome-wide scale has been generated for different anatomical segments of normal and AD brains. Thus, our study creates a useful data set supplementing previous microarray-generated information, which lacked isoform-specific resolution of gene expression [9], [41].

Despite the magnitude of the *APOE* e4 risk effect and a possible mechanistic link with amyloid beta (Aβ) pathology [34], [42], [43], it is still far from clear how *APOE* e4 is involved in AD pathogenesis [44]. Interestingly, the *APOE* genotype in the case of AD samples used in this study was e3, which is considered to have no effect on AD onset. This suggests that the observed alternative promoter and TSS usage during *APOE* expression in the AD temporal lobe might be independent of the Cys⇒Arg substitution at position 112. Following this line of reasoning, differential *APOE* expression patterns - as indicated in this report - might be independent of the amyloid beta aggregation pathway in the course of Alzheimer's disease. Indeed, previous observations of alternative splicing in AD brains for glutamate transporter [45], *PIN1* [46], estrogen receptor alpha [47] and the *APOE* receptor [48] genes strongly suggest that alteration of transcriptional control for genes involved in neuronal physiology is a landmark of ongoing neurodegeneration. In light of our observations of alternative *APOE* expression, the previously reported AD-specific splicing pattern of the *APOE* receptor further suggests the functional relevance of lipid metabolism in the context of AD pathology [49]. Moreover, it has previously been proposed that synthesis of ApoE might play a role in regional vulnerability of neurons in AD [50]. How this might relate to the presence of different transcriptional variants of *APOE* remains a subject for future studies.

## Supporting Information

Table S1 Top 30 up and top 30 down regulated genes in AD total brain. Differential gene expression for total brain was calculated using the ratio of AD versus normal FPKM values for every gene identified as expressed by Cufflinks. The genes were ranked on this ratio (fold change), and those with the 30 highest and 30 lowest fold change values are shown here.
(XLSX)

Table S2 Top 30 up and top 30 down regulated genes in AD frontal lobe. Differential gene expression for frontal lobe was calculated using the ratio of AD versus normal FPKM values for every gene identified as expressed by Cufflinks. The genes were ranked on this ratio (fold change), and those with the 30 highest and 30 lowest fold change values are shown here.
(XLSX)

Table S3 Top 30 up and top 30 down regulated genes in AD temporal lobe. Differential gene expression for temporal lobe was calculated using the ratio of AD versus normal FPKM values for every gene identified as expressed by Cufflinks. The genes were ranked on this ratio (fold change), and those with the 30 highest and 30 lowest fold change values are shown here.
(XLSX)

Table S4 Top 20 Clusters from functional enrichment analysis using the DAVID tool for total brain. The NCBI tool, DAVID, was used to investigate functional associations of gene expression changes seen in AD total brain. There were 1071 genes that were more than two-fold over- or under-expressed in AD relative to normal total brain and these were analysed by the functional clustering tool. Gene Ontology Biological Process was selected as the annotation category for clustering. Once the tool has identified enriched ontologies for a particular gene list, it creates annotation clusters with those that have a statistically significant overlap in terms of their constituent genes. The top 20 annotation clusters are shown in this table.
(XLSX)

Table S5 Top 20 Clusters from functional enrichment analysis using the DAVID tool for frontal lobe. The NCBI tool, DAVID, was used to investigate functional associations of gene expression changes seen in AD frontal lobe. There were 944 genes that were more than two-fold over- or under-expressed in AD relative to normal frontal lobe and these were analysed by the functional clustering tool. Gene Ontology Biological Process was selected as the annotation category for clustering. Once the tool has identified enriched ontologies for a particular gene list, it creates annotation clusters with those that have a statistically significant overlap in terms of their constituent genes. The top 20 annotation clusters are shown in this table.
(XLSX)

Table S6 Top 20 Clusters from functional enrichment analysis using the DAVID tool for temporal lobe. The NCBI tool, DAVID, was used to investigate functional associations of gene expression changes seen in AD temporal lobe. There were 1416 genes that were more than two-fold over- or under-expressed in AD relative to normal temporal lobe and these were analysed by the functional clustering tool. Gene Ontology Biological Process was selected as the annotation category for clustering. Once the tool has identified enriched ontologies for a particular gene list, it creates annotation clusters with those that have a statistically significant overlap in terms of their constituent genes. The top 20 annotation clusters are shown in this table.
(XLSX)
